# In vitro immuno‐prevention of nitration/dysfunction of myogenic stem cell activator HGF, towards developing a strategy for age‐related muscle atrophy

**DOI:** 10.1111/acel.14337

**Published:** 2024-09-19

**Authors:** Sakiho Tanaka, Alaa Elgaabari, Miyumi Seki, So Kuwakado, Kahona Zushi, Junri Miyamoto, Shoko Sawano, Wataru Mizunoya, Kenshiro Ehara, Naruha Watanabe, Yohei Ogawa, Hikaru Imakyure, Reina Fujimaru, Rika Osaki, Kazuki Shitamitsu, Kaoru Mizoguchi, Tomoki Ushijima, Takahiro Maeno, Takashi Nakashima, Takahiro Suzuki, Mako Nakamura, Judy E. Anderson, Ryuichi Tatsumi

**Affiliations:** ^1^ Department of Animal and Marine Bioresource Sciences Graduate School of Agriculture, Kyushu University Fukuoka Japan; ^2^ Department of Physiology, Faculty of Veterinary Medicine Kafrelsheikh University Kafrelsheikh Egypt; ^3^ Department of Orthopaedic Surgery, Faculty of Medical Sciences Kyushu University Fukuoka Japan; ^4^ Department of Food and Life Science, School of Life and Environmental Science Azabu University Sagamihara Japan; ^5^ Department of Animal Science and Biotechnology, School of Veterinary Medicine Azabu University Sagamihara Japan; ^6^ Department of Bioscience and Biotechnology, Graduate School of Agriculture Kyushu University Fukuoka Japan; ^7^ Department of Biological Sciences, Faculty of Science University of Manitoba Winnipeg Manitoba Canada

**Keywords:** c‐met binding, hepatocyte growth factor (HGF), monoclonal antibody, muscle atrophy, peroxynitrite, rat, resident myogenic stem satellite cell, tyrosine nitration

## Abstract

In response to peroxynitrite (ONOO^−^) generation, myogenic stem satellite cell activator HGF (hepatocyte growth factor) undergoes nitration of tyrosine residues (Y198 and Y250) predominantly on fast IIa and IIx myofibers to lose its binding to the signaling receptor c‐met, thereby disturbing muscle homeostasis during aging. Here we show that rat anti‐HGF monoclonal antibody (mAb) 1H41C10, which was raised in‐house against a synthetic peptide FTSNPEVR_nitro_Y_198_EV, a site well‐conserved in mammals, functions to confer resistance to nitration dysfunction on HGF. 1H41C10 was characterized by recognizing both nitrated and non‐nitrated HGF with different affinities as revealed by Western blotting, indicating that the paratope of 1H41C10 may bind to the immediate vicinity of Y198. Subsequent experiments showed that 1H41C10‐bound HGF resists peroxynitrite‐induced nitration of Y198. A companion mAb‐1H42F4 presented similar immuno‐reactivity, but did not protect Y198 nitration, and thus served as the control. Importantly, 1H41C10‐HGF also withstood Y250 nitration to retain c‐met binding and satellite cell activation functions in culture. The Fab region of 1H41C10 exerts resistivity to Y250 nitration possibly due to its localization in the immediate vicinity to Y250, as supported by an additional set of experiments showing that the 1H41C10‐Fab confers Y250‐nitration resistance which the Fc segment does not. Findings highlight the in vitro preventive impact of 1H41C10 on HGF nitration‐dysfunction that strongly impairs myogenic stem cell dynamics, potentially pioneering cogent strategies for counteracting or treating age‐related muscle atrophy with fibrosis (including sarcopenia and frailty) and the therapeutic application of investigational HGF drugs.

## Graphical abstract

Previously, we demonstrated age‐related nitration of ECM‐bound HGF (myogenic stem satellite cell activator), preferentially in fast IIa and IIx myofibers. Rat anti‐HGF monoclonal antibody 1C10 was generated; 1C10‐bound HGF resists peroxynitrite‐induced nitration of HGF‐Y198/Y250 to retain its c‐met binding and satellite cell activation activities in vitro. 1C10‐Fab region confers nitration dysfunction resistance to HGF. This discovery potentially pioneers cogent strategies for counteracting or treating age‐related muscle atrophy with fibrosis (including sarcopenia and frailty) and the therapeutic application of investigational HGF drugs.

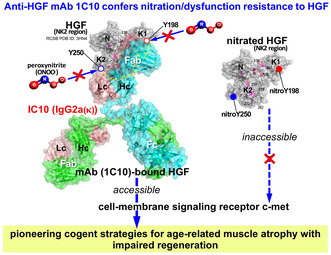

**Abbreviations**
BrdU: 5‐bromo‐2′‐deoxyuridine
BSA: bovine serum albumin
DAB: 3,3′‐diaminobenzidine
DMEM: Dulbecco's modified Eagle's medium
ECL: enhanced chemiluminescence
Fab: fragment antigen‐binding
Fc: fragment crystallizable
HGF: hepatocyte growth factor
HRP: horseradish peroxidase
HS: horse serum
mAb: monoclonal antibody
SDS: sodium dodecyl sulfate
TTBS: tris‐buffered saline
Y: tyrosine residue
βME: β‐mercaptoethanol

Several studies have demonstrated that HGF is a pleiotropic cytokine, displaying critical functions for development, regeneration, and repair of a variety of tissues and organs including skeletal muscle, liver, kidney, lung, brain, and bone, by promoting cell activation, migration, proliferation, and survival (from apoptosis and necrosis) that mediate myogenesis, angiogenesis, neuritogenesis, and fibrogenesis (Anderson et al., 2017; Desole et al., 2021; Honda et al., 1995; Kawaida et al., 1994; Lee et al., 2022; Masuda et al., 2015; Matsumoto et al., 2014; Nakamura, 1994; Ohmichi & Matsumoto, 1996; Panganiban & Day, 2011; Park et al., 2022; Tatsumi et al., 2017; Tatsumi, Sankoda, et al., 2009; Wang et al., 2018; Yun et al., 2012; Zhang et al., 2020; Zhao et al., 2022). Therapeutic applications of HGF have been tested in various diseases including liver cirrhosis, chronic renal failure, lung fibrosis, myocardial infarction, arteriosclerosis obliterans, amyotrophic lateral sclerosis, and acute spinal cord injury (Matsuda, 1997; Matsumoto, 2002; Matsumoto & Nakamura, 1998; Morishita et al., 2004; Powell et al., 2010; Sala & Crepaldi, 2011; Sanada et al., 2020; Takano et al., 2017; Ueki et al., 1999).

Our previous studies revealed the essential role of HGF in postnatal muscle growth and regeneration by demonstrating that myogenic stem satellite cell activation is triggered by mechanical perturbation through a cascade of events including release of the active form of HGF (hetero‐dimer of α‐ and β‐chains) from its extracellular tethering in a nitric oxide (NO) radical‐dependent manner, and the subsequent presentation of HGF to the cell membrane signaling receptor c‐met (Allen et al., 1995; Anderson, 2000; Hara et al., 2012; Tatsumi, 2010; Tatsumi et al., 1998, 2001, 2002, 2006, 2009; Tatsumi & Allen, 2004, 2008; Yamada et al., 2006, 2008). Importantly, recent direct‐immunofluorescence microscopy of rat lower hindlimb muscles revealed that with aging, extracellular matrix (ECM)‐bound HGF undergoes tyrosine residue (Y) nitration‐dysfunction (Elgaabari et al., 2024), a posttranslational chemical modification process for the nonenzymatic introduction of a nitro group (‐NO_2_) into the side chain by peroxynitrite (ONOO^−^, a short‐lived biomolecule generated by the rapid reaction of NO with superoxide radicals in vivo), thereby disturbing muscle homeostasis. Protein tyrosine nitration is a highly selective event (Ischiropoulos, 2009; Viner et al., 1996) and detrimental accumulation of specifically nitrated proteins has been observed in a variety of disorders including aging (Fugere et al., 2006; Pandya et al., 2019) and Alzheimer's and Parkinson's diseases (Good et al., 1998; Horiguchi et al., 2003; Reyes et al., 2008).

Subsequent biochemical and immunochemical studies demonstrated that the HGF nitration takes place at Y198 and Y250, characterized by their respective locations at the c‐met binding sites of the K1 and K2 domains of HGF α‐chain. Such nitration‐caused dysfunction is specific to HGF among the other major growth factors examined. Age‐related increases in nitration of ECM‐bound HGF was predominant on fast IIa and IIx myofibers, as visualized by anti‐nitrated Y198‐ and Y250‐HGF monoclonal antibodies (mAbs, rat clones 3A11C6, and 6B82C3 raised in‐house, respectively) in experiments employing three age groups of young, adult, and old rats, highlighting inhibitory impacts of accumulation of nitrated HGF on myogenic stem cell dynamics (Elgaabari et al., 2024). Prevention of HGF nitration dysfunction is novel for its potential to suppress the progression of age‐related disorders of muscle homeostasis including muscle atrophy and impaired regeneration with intramuscular fibrosis and also to improve the therapeutic effects of investigational HGF drugs.

In this study, as a possible molecular tool to inhibit HGF nitration, we looked to the mAb, which recently gained attention as a therapeutic with high specificity and affinity for narrow epitope sequences of antigens and hence selective drug delivery to target tissues and organs (Zahavi & Weiner, 2020). In the screening and cloning of the rat anti‐nitrated Y198‐HGF mAb 3A11C6 described above, two additional clones 1H41C10 and 1H42F4 were obtained (see Figure S1; abbreviated as 1C10 and 2F4, respectively; IgG2a(κ) immunoglobulin isotype) and characterized for their immuno‐reactivities to nitrated HGF and non‐nitrated HGF (recombinant mouse HGF exposed to peroxynitrite at pH 7.4) by ECL Western blotting as shown in Figure 1a,b. Loading amounts of HGF α‐chain on the blot were comparable between peroxynitrite‐treated and untreated control lanes, as revealed by stripping with SDS‐βME solution and re‐probing the membrane with anti‐HGF *N*‐terminal domain mAb. Results clearly showed that 1C10 and 2F4 mAbs bind to both non‐nitrated and nitrated HGF (the 90‐kDa proform not yet cleaved into α‐ and β‐chains, and 60‐kDa α‐chain) with significantly different affinity under reducing conditions (non‐nitrated HGF < nitrated HGF) (Figure 1b, right column). The immuno‐reactivity observed was in contrast to that of the anti‐nitroY198‐HGF specific mAb 1C6 that was previously characterized (Elgaabari et al., 2024) and hence served as the control mAb here (see Figure 1b, left column). These features indicate that the paratope (*N*‐terminal tip of the fragment antigen‐binding (Fab) region) of 1C10 and 2F4 mAbs may bind to the immediate vicinity of Y198 in the nitration site of HGF in a unique manner that allows paratope recognition of the nitration status of Y198.

**FIGURE 1:**
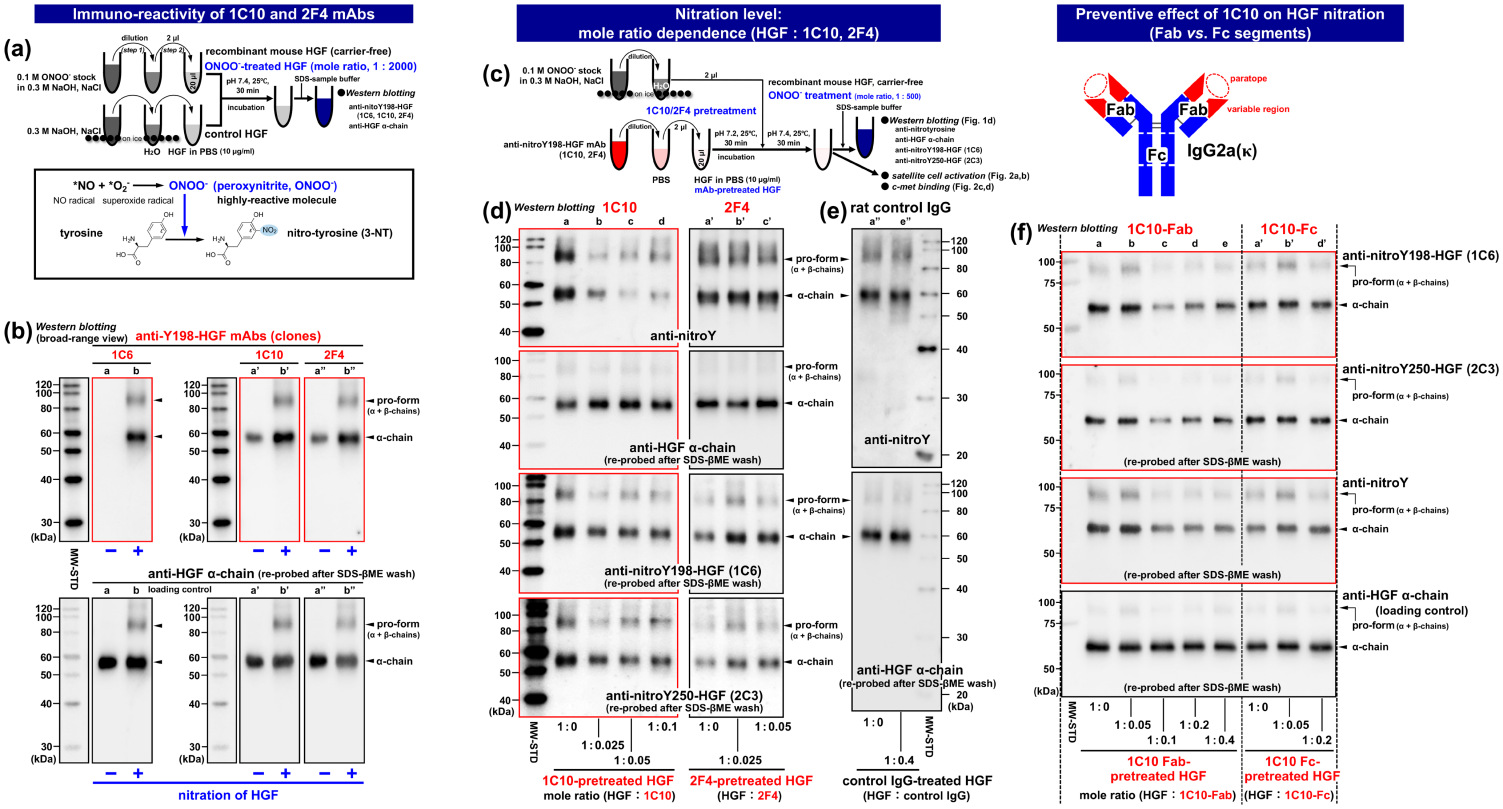
Effect of pretreatment with anti‐Y198‐HGF mAbs 1C10 and 2F4 on HGF nitration. (a) In vitro experimental design for the preparation of nitrated and non‐nitrated HGF. Two‐step dilution protocol for the peroxynitrite stock (0.1 M ONOO^−^ on ice) was optimized for the exposure of recombinant HGF (carrier protein‐free; disulfide‐linked heterodimer of 60‐kDa α‐chain and 30‐kDa β‐chain as the major form in the product) to active peroxynitrite at 1:2000 (mole ratio relative to HGF), pH 7.4, 25°C, for 30 min, in order to induce nitration of Y198 and Y250 under physiological conditions, as described previously (Elgaabari et al., 2024). Control HGF (non‐nitrated HGF), incubated in treatment buffer at pH 7.4 only, without peroxynitrite for 30 min. Lower column is a supplemental sketch of tyrosine residue nitration, peroxynitrite‐induced nitrotyrosine formation by introduction of a nitro group (‐NO_2_) into a Cε atom in aromatic ring of the side chain. (b) Characterization of anti‐Y198‐HGF mAbs 1C10 and 2F4. Visualization of immuno‐reactivity of anti‐Y198‐HGF mAbs IH41C10 and IH42F4 (abbreviated as 1C10 and 2F4, respectively) by ECL Western blotting of non‐nitrated control HGF (lanes a’ and a”) and nitrated HGF (lanes b’ and b”). Blots were first treated with 1C10/2F4 and HRP‐conjugated anti‐rat IgG Abs (upper column), followed by re‐probing with HRP‐labeled anti‐HGF α‐chain mAb after stripping with SDS‐βME solution (lower column). Hybridoma clone 3A11C6 (abbreviated as 1C6; raised and characterized by Elgaabari et al., 2024) served as a control and displayed high immuno‐specificity to nitroY198‐HGF (lanes a and b). A wide molecular weight range (30–120 kDa) covers the 90‐kDa HGF proform and the 60‐kDa HGF α‐chain (including the NK2 segment responsible for c‐met binding) was displayed. MW‐STD, MagicMark molecular weight standards. (c) Experimental design for evaluating the effect of pretreatment with anti‐Y198‐HGF mAbs 1C10 and 2F4 on HGF nitration. Recombinant HGF was incubated with 1C10 and 2F4 in a range of 1:0–1:0.1 (mole ratios relative to HGF) for 30 min prior to peroxynitrite protocol (at 1:500 relative to HGF, pH 7.4, 25°C, for 30 min). (d) Evaluation of nitration levels of HGF pretreated with 1C10 (lanes a–d) and 2F4 (lanes a’–c’). Assayed by HRPO‐labeled anti‐nitrotyrosine (anti‐nitroY) mAb in Western blotting format normalized by total HGF α‐chain (detected with HRP‐labeled anti‐HGF α‐chain mAb after the stripping step as described in Figure 1b), followed by re‐probing with anti‐nitroY198‐HGF mAb (clone 1C6) and anti‐nitroY250‐HGF mAb (clone 2C3); clones 1C6 and 2C3 mAbs displayed high immuno‐specificity to nitroY198‐HGF and nitroY250‐HGF, respectively, as described previously (Elgaabari et al., 2024). Densitometric analysis of panel d, normalized with total HGF α‐chain (positive for anti‐HGF α‐chain mAb) is shown in Figure S2. (e) Evaluation of nitration levels of HGF pretreated with control IgG (1:0.4 mole ratio relative to HGF). Assayed by western blotting normalized by whole HGF as same as panel d (lanes a” and e”). MW‐STD, MagicMark molecular weight standards. Note that there was no detectable effect of control IgG even at 1:0.4 that is higher mole ratio than the 1C10/2F4 treatments (see panel d). (f) Evaluation of preventive effect on HGF‐Y198/Y250 nitration (Fab vs. Fc fragments of 1C10 IgG). Nitration levels of HGF pretreated with 1C10‐Fab (lanes a–e) and 1C10‐Fc (lanes a’, b’, and d). Assayed by anti‐nitroY198‐HGF mAb (1C6; *first row*) and anti‐nitroY250‐HGF mAb (2C3; *second row*) and HRP‐labeled anti‐rat IgG‐Fc secondary Ab, and then by HRPO‐labeled anti‐nitrotyrosine mAb (anti‐nitroY; *third row*) in Western blotting format normalized by total HGF α‐chain (detected with HRP‐labeled anti‐HGF α‐chain mAb; *fourth row*) as described in Figure 1d. MW‐STD, ProteinLadder molecular weight standards.

Given the possible localization of 1C10 and 2F4 mAb epitopes as described above, further experiments were designed to examine if these mAbs have potential for preventing peroxynitrite‐induced nitration‐dysfunction of HGF (Figures 1c–f and 2a,b). When recombinant HGF was pretreated with 1C10 at pH 7.2 for 30 min prior to exposure to peroxynitrite (1:500 of a mole ratio of peroxynitrite relative to HGF), the tyrosine nitration level was reduced in proportion to the mole ratio of 1C10 to HGF, as revealed by Western blotting. Blotting showed a decrease in anti‐nitroY mAb‐positive HGF (60‐kDa α‐chain and 90‐kDa proform) normalized with total HGF α‐chain as a loading control (Figure 1d, first and second rows, lanes a–d). A comparable outcome was documented in instances of elevated peroxynitrite exposure (1:2000 mole ratio relative to HGF (Figure S5, lanes a–e), which provides encouraging evidence for the preventive effect of 1C10 on peroxynitrite‐induced nitration of HGF. Importantly, decreased Y198 nitration was visualized by re‐probing the blots with 1C6 anti‐nitroY198‐HGF‐specific mAb (Figure 1d, third row, lanes a–d). Companion mAb 2F4 was evaluated in the same manner, and displayed no significant immuno‐preventive effect on HGF nitration detected by anti‐nitroY and 1C6 anti‐nitroY198‐HGF mAbs, and hence served as the control IgG2a(κ) here (Figure 1d, lanes a'–c', and Figure S5, lanes a'–d'). The result was further verified by an additional set of experiments using rat control IgG (Figure 1e, lanes a" and e"), in which there was no observed effect on the nitration level even at 1:0.4, a higher mole ratio than the above 1C10 and 2F4 treatments (1:0.1 and 1:0.05, respectively; see panel d). These results encourage the emerging conclusion that 1C10‐bound HGF displays resistance to Y198‐nitration in vitro.

**FIGURE 2:**
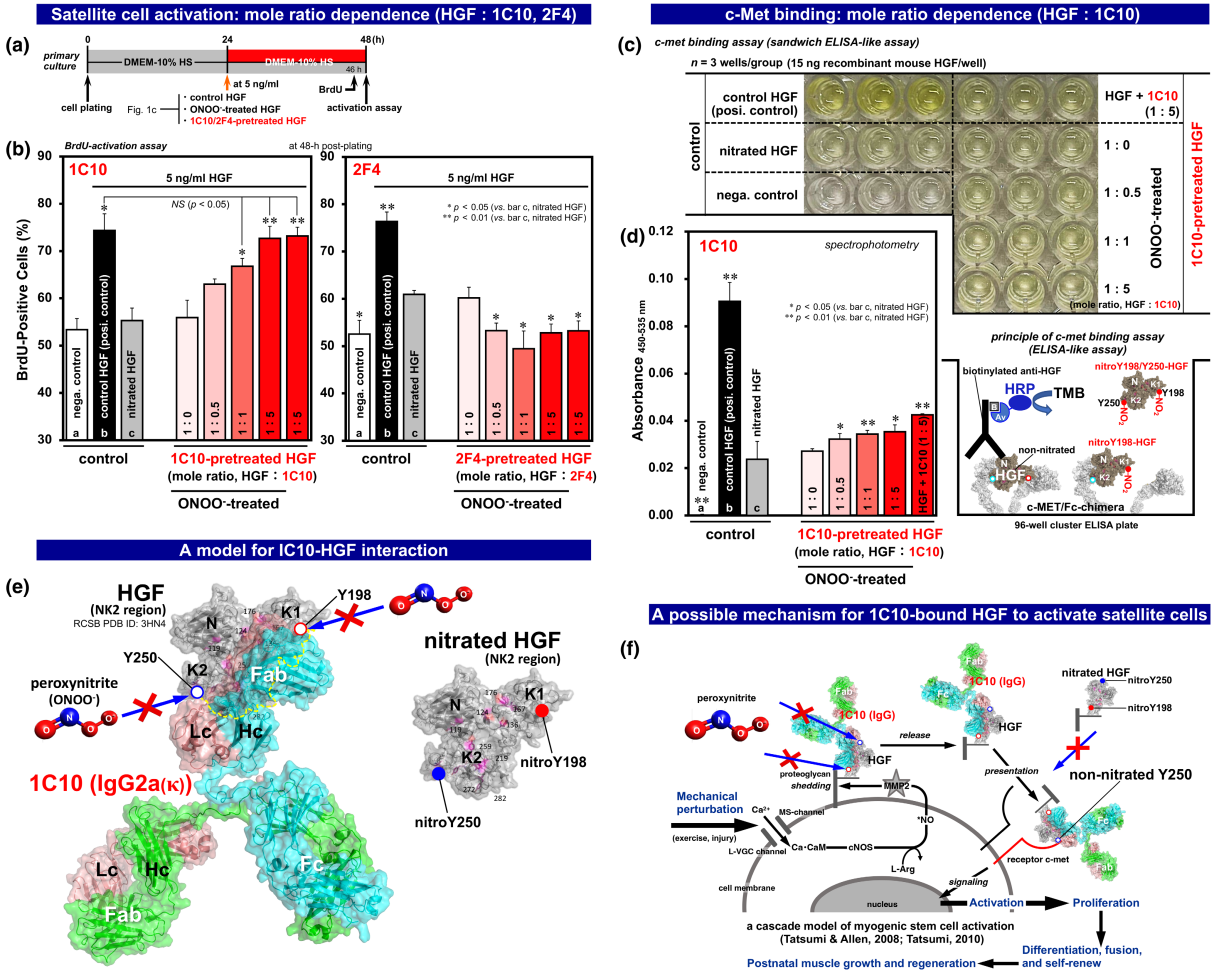
Prevention of HGF nitration‐dysfunction by interaction with anti‐Y198‐HGF mAb 1C10. (a) The experimental scheme for assays in culture. Primary cultures of rat satellite cells received 5 ng/mL HGF (control, peroxynitrite‐treated, and 1C10/2F4‐pretreated HGF prior to the peroxynitrite treatment, as shown in Figure 1c,d) in DMEM‐10% HS (pH 7.2) for 24 h beginning at 24‐h post‐plating followed by pulse‐labeling with BrdU for 2 h just prior to the 48‐h, time‐point of the BrdU‐incorporation assay for activation. (b) Cell activation response to HGF that was pretreated with 1C10 and 2F4 before exposure to peroxynitrite (ONOO^−^). Satellite cell cultures were assayed for cell activation (BrdU‐positive cell percentage) at 48‐h post‐plating. *Open bars a*, negative control untreated‐cultures in DMEM‐10% HS; *black‐solid bars b*, positive control cultures with 5 ng/mL recombinant HGF for 24 h beginning 24‐h post‐plating; *gray bars c*, cultures with 5 ng/mL nitrated HGF (peroxynitrite‐treated at 1:500 relative to HGF). *Red‐gradient bars*, cultures with 5 ng/mL HGF that received pretreatment with 1C10 and 2F4 (at 1:0, 1:0.5, 1:1, and 1:5 mole ratios relative to HGF) prior to the peroxynitrite treatment. *Far‐right red‐bars*, cultures with 5 ng/mL HGF that just received the 1C10/2F4 pretreatment (at 1:5, without peroxynitrite treatment). Bars represent mean ± SEM and significant differences from the nitrated HGF (*bars c*) at *p <* 0.05 and *p <* 0.01 are indicated by (*) and (**), respectively. *NS*, not significant at *p <* 0.05. (c) Sandwich ELISA‐like assay on recombinant c‐met‐Fc chimera (see photo of the triplicate assay and schematic diagram of the mechanism of action). (d) Receptor c‐met binding activity of HGF pretreated with mAb 1C10. Optical absorbance_450‐535 nm_ was presented by subtracting the value of the negative control without HGF (*bar a*). *Black bar b*, control HGF without any treatment served as a positive control; *gray bar c*, nitrated HGF (1:500 peroxynitrite treatment); *red‐gradient bars* (*from left to right*), HGF pretreated with 1C10 (at 1:0, 1:0.5, 1:1, and 1:5 mole ratios relative to HGF) prior to 1:500 peroxynitrite treatment. *Far‐right red bar*, HGF that just received the 1C10 pretreatment (at 1:5, without peroxynitrite). Bars represent mean ± SEM and significant differences from the nitrated HGF (*bar c*) at *p <* 0.05 and *p <* 0.01 are indicated by (*) and (**), respectively. (e) A possible model for interaction of HGF (NK2 region composed of N, K1, and K2 domains; RCSB PDB ID: 3HN4 (Tolbert et al., 2010) and 1C10 mAb. A paratope of 1C10 IgG2a(κ), also known as an antigen‐binding site (a small region at *N*‐terminal tips of the heavy (Hc) and light chains (Lc) in Fab region), binds to a Y198‐containing epitope in K1 domain to prevent peroxynitrite‐induced nitration of Y198 (indicated by *red open‐circle*). Fab may also contribute to the resistivity of Y250 to nitration, possibly by localizing very close to Y250 (non‐nitrated form; indicated by *blue open circle*); the area hidden behind the 1C10‐Fab is outlined with a yellow broken line. The three‐dimensional structure of IgG2a(κ) was re‐drawn from synchrotron diffraction data for mouse anti‐malignant canine lymphoma mAb (RCSB PDB ID: 1IGT, with 2.80 Å resolution (Harris et al., 1997) using the program PyMOL Molecular Graphics System (Version 2.6.0a0; copyright: Schrödinger, LLC., created by Dr. Warren L. DeLano). Crystal structure of human HGF‐NK2 segment, originally displayed based on RCSB PDB ID 3HN4 (Tolbert et al., 2010) and reproduced from Elgaabari et al. (2024) with pink‐highlighting all tyrosine residues including Y198 and Y250 indicated by red and blue circles, respectively. (f) Schematic presentation of a possible mechanism for satellite cell activation by 1C10‐bound HGF. Quiescent satellite cells are activated to re‐enter the cell cycle in response to mechanical perturbation of muscle tissue through a molecular cascade of events including calcium ion influx from extracellular compartment by functional coupling of a mechano‐sensitive cation channel (MS‐channel) and a long‐lasting type of voltage‐gated Ca^2+^ channel (L‐VGC channel), calcium‐calmodulin (Ca‐CaM) formation, NO radical production by activated constitutive NO synthase (cNOS; neuronal NOS and endothelial NOS), matrix metalloproteinase 2 (MMP2) activation, rapid release of HGF from its extracellular tethering (possibly with associated extracellular segment of proteoglycans) to give rise to ng/ml level of the growth factor, and the subsequent presentation to cell‐membrane receptor c‐met to generate a signal for satellite cell activation (reproduced from Tatsumi & Allen, 2008 and Tatsumi, 2010) with some modifications). 1C10 binds to ECM‐bound HGF as revealed by immuno‐fluorescence staining of satellite cell culture (Figure S4 panel c, visualized at 24‐h and 48‐h post‐plating). The affinity of the HGF‐1C10 complex to c‐met is lower than that of HGF ligand alone because: (i) both Y198 and Y250 localize in the c‐met binding sites as revealed by the cryo‐EM structure of a complex of HGF and the receptor c‐met dimer (RCSB PDB ID: 7MO7) (Uchikawa et al., 2021); (ii) Y198 is occupied by 1C10‐Fab, making the K1 domain of HGF inaccessible to c‐met; and (iii) Y250 is prevented from nitration possibly through the steric hindrance action of IC10‐Fab as modeled in panel e and hence able to associate with c‐met monomer to generate an activation signal as a result (see a c‐met depicted by the red line in panel f, redrawn to focus on the binding of HGF‐1C10 complex to c‐met monomer in contrast to nitroY198, 250‐HGF that does not bind to c‐met).

Additionally, 1C10 also works for resisting nitration of Y250‐HGF, as shown in Figure 1d (fourth row, lanes a–d), demonstrated by re‐probing the blots with 2C3 anti‐nitroY250‐HGF specific mAb (also see densitometric analysis shown in Figure S2 left column). To provide a logical interpretation for the unexpected observation, we had an insight that either the Fab or fragment‐crystallizable (Fc) region of 1C10 IgG may be involved in resisting Y250 nitration. As shown in Figure 1f (lanes a–e), when the Fc region was removed from 1C10 IgG (composed of two Fab and one Fc segments) by papain digestion and protein‐L affinity chromatography, the resulting 1C10‐Fab exhibited the usual activity to prevent tyrosine‐residue nitration in a mole ratio‐dependent manner (from 1:0.05 to 1:0.4) as revealed by Western blotting of anti‐nitroY mAb (third row). The immuno‐prevention of Y198 and Y250 nitration was visualized by the 1C6 anti‐nitroY198‐HGF (first row) and 2C3 anti‐nitroY250‐HGF mAbs (second row), respectively, supporting the hypothesis concerned. Considering the additional result, namely that isolated 1C10‐Fc did not show any preventive effect on nitration of Y198 and Y250 in the examined mole ratio range from 1:0.05 to 1:0.2 (Figure 1f, lanes a’, b’, and d’), the 1C10‐Fab segment is interpreted as being solely responsible for the biochemical function of Y250‐nitration resistance (see Figure 2e for better understanding a possible configuration).

The therapeutic availability of the characterized 1C10 mAb was investigated by evaluating the myogenic stem satellite cell activation activity of 1C10‐bound HGF in culture (Figure 2a,b). Primary cultures of satellite cells were prepared from the upper hindlimb and back muscles of adult rats (Allen et al., 1997) and cultured for 24 h beginning at 24‐h post‐plating in DMEM‐10% HS containing 5 ng/mL HGF that was pretreated with 1C10 at mole ratios from 1:0.5 to 1:5 just prior to peroxynitrite treatment. The activation index in each condition was evaluated by determining the percentage of BrdU‐positive cells, indicative of the cell activation response to HGF samples (Figure 2b, left column, *red‐gradient bars*). Results clearly showed that the activation index was rescued further at higher 1C10 mole ratios (1:1 and 1:5), up to a level comparable to a positive control culture (*bar b*) exposed to untreated HGF at the 5 ng/mL dose that maximizes satellite cell activation in culture (Tatsumi et al., 1998). Nitrated HGF (*bar c*, peroxynitrite‐treated) lost its activation activity as demonstrated by a drop in the activation index down to the baseline level observed in the negative control culture treated only with medium lacking HGF (*bar a*), consistent with the previous report (Elgaabari et al., 2024). These experiments provide a robust platform demonstrating the unique functionality of the 1C10 mAb that prevents nitration‐dysfunction of the myogenic stem cell activator HGF. In contrast to 1C10, the companion mAb 2F4, did not display a significant increase in the activation index (BrdU‐positive cell percentage) after treating satellite‐cell cultures in the same format, over the same range of mole ratios as for 1C10 (Figure 2b, right column, *red‐gradient bars*). These observations are consistent with the null‐preventive effect of 2F4 on HGF nitration illustrated earlier by Western blotting (Figure 1d, lanes a’–c’). Results highlight the unique preventive function of the 1C10 mAb in peroxynitrite‐induced Y198/Y250‐nitration/dysfunction of HGF to guarantee satellite cell activation activity of 1C10‐bound HGF.

This novel finding was further encouraged by c‐met binding experiments, in which HGF was pretreated with 1C10 followed by exposure to peroxynitrite and evaluated for its c‐met binding by sandwich ELISA‐like assay on recombinant c‐met‐Fc chimera (Figure 2c,d). The assay was designed to demonstrate the biochemical activity of c‐met binding sites including Y198 and Y250 (in K1 and K2 domains of HGF, respectively) by significant changes in optical absorbance within a range from a baseline level (nitrated HGF; panel d, *bar c*) to a positive control (non‐nitrated HGF; *bar b*). Binding affinity of the 1C10‐bound HGF was elevated from the baseline level in proportion with an increasing mole ratio of 1C10 relative to HGF (*red‐gradient bars*).

Interestingly, even at mole ratios of 1:1 and 1:5, at which the satellite‐cell activation index was rescued to a level comparable to that of non‐nitrated “regular” HGF as shown previously (Figure 2b, left column), c‐met binding affinity only reached approximately 20% of the full activity of the positive control. This result is reasonable from the following perspectives. First, the maximum affinity of the receptor c‐met for HGF requires both Y198 and Y250 to be non‐nitrated, which constitute the respective c‐met binding sites in K1 and K2 domains (Elgaabari et al., 2022, 2024; Uchikawa et al., 2021), as demonstrated again in Figure S3 showing that the binding affinity of the non‐nitrated NK1 segment (lacking Y250) for c‐met is about 15% of that of HGF at the same molar concentration (0.015 μM) (Elgaabari et al., 2022). Second, the association or localization of about 50‐kDa 1C10‐Fab region in the vicinity of the non‐nitrated Y198‐K1 domain may partly hinder the structural presentation of HGF to the receptor c‐met. Third, the Y250‐K2 domain is also prevented from nitration (as shown in Figure 1d, left column) possibly through the steric hindrance‐like action of IC10‐Fab as modeled in Figure 2e, and hence available to binding to a single c‐met molecule that generates signals to activate quiescent satellite cells without dimerization of c‐met molecules that typically sandwich a HGF ligand (see a model shown in Figure 2f). This insight is supported by the observation that the culture with 5 ng/mL HGF, which received only the 1C10 pretreatment (at 1:5, without peroxynitrite treatment), exhibited indices of satellite‐cell activation (% BrdU‐positive cells assayed at 48‐h post‐plating) and subsequent proliferation (evaluated by mRNA expression levels of MyoD and its counter‐regulatory factor myogenin at 24–72 h time‐points) equivalent to that of the positive control culture treated with 5 ng/mL regular HGF without nitration (Figure 2b left column, *far‐right red bar* and *bar b*, and Figure S4, respectively).

In conclusion, we demonstrated that the rat 1C10 IgG2a(κ) raised against a nitrated Y198‐HGF peptide (FTSNPEVRY_198_ EV, well‐conserved in mammals) effectively prevents nitration of Y198 and Y250, thereby preserving the ability of HGF to activate myogenic stem satellite cells in vitro (Figures 1d and 2b). The immuno‐preventive effect is attributed to the Fab region of 1C10 (Figure 1f). 1C10‐bound HGF binds to the receptor c‐met with a lower affinity than regular HGF possibly through interaction with a Y250‐containing site in the K2 domain (not a Y198‐containing K1 domain) to generate a normal activation signal in vitro as shown in Figure 2a–d; a smaller 1C10‐Fab segment (i.e., without an attached Fc fragment) would be expected to further enhance the affinity and distribution (and possibly reduce side effects if used in a therapeutic application), expanding its applicability and development potential for therapeutic antibodies.

The present work encourages additional research on the first potential immuno‐preventative application of extracellular HGF nitration‐dysfunction. Further exploration could lead to strategies for its use in age‐related atrophy, although at this stage we have not provided evidence that mAb 1C10 can mitigate sarcopenia or that it has therapeutic value, awaiting the results of in vivo experiments. This mechanism potentially has promise for use in developing new pharmaceutical strategies to maintain HGF functions in many health conditions. In particular, combating age‐related muscle atrophy with impaired regeneration (including sarcopenia and frailty), could extend the expectation of sustained muscle health into old age by improving human, animal, and pet welfare and sustaining food security through meat animal production. Importantly, HGF displays important and pleiotropic functions in a variety of tissues and organs and therefore therapeutic applications of HGF have been tested in various diseases as described above. In vitro immuno‐prevention of HGF nitration‐dysfunction may be an important foundational perspective from which improve prospects for the therapeutic effectiveness of investigational HGF drugs as well as their effective tissue‐specific delivery. This strategy may be adaptable to several disorders and diseases that show the generation and accumulation of particular nitrated proteins at specific sites, including cardiovascular damage (Mihm et al., 2001; Shishehbor et al., 2003), chronic obstructive pulmonary disease (COPD) (Barreiro et al., 2003), colon and lung cancer (Gochman et al., 2011; Markowitz et al., 2017; Zhan et al., 2018), Alzheimer's and Parkinson's diseases (Castegna et al., 2003; Good et al., 1998; He et al., 2019; Horiguchi et al., 2003; Reyes et al., 2008), and aging (Chakravarti & Chakravarti, 2017; Elgaabari et al., 2024; Fugere et al., 2006; Kanski et al., 2005; Prolo et al., 2024; Viner et al., 1996) although the direct causal relationship remains unclear and awaits further studies.

## AUTHOR CONTRIBUTIONS

R. T. conceived the project. S. T., A. E., and R. T. designed the experiments. S. T., A. E., and R. T. performed the experiments. T. N. worked on the 3D model of the IgG2a(κ)‐Fab region accessible to Y250‐HGF and its graphical representation. S. T., A. E., M. S., S. K., K. Z., T. N., T. S., J. E. A., and R. T. analyzed the data. J. M., S. S., W. M., K. E., T. M., T. N., T. S., and M.K. contributed reagents, materials, and/or analysis tools and provided advice on project progress and technical help. K. Z., N. W., Y. O., H. I., R. F., R.O., K. S., K. M., T. U., T. S., and R. T. performed additional experiments shown in Figure S4. S. T., A. E., J. E. A., and R. T. wrote the manuscript.

## FUNDING INFORMATION

This work was supported by Grants‐in‐Aid for Challenging Exploratory Research, Scientific Research (B), and International Research Fellow from the Japan Society for the Promotion of Science (JSPS KAKENHI grant numbers JP26660218, JP21H02347 and JP24K01911, and JP22F22389, respectively) (all to R. Tatsumi). The research was also supported in part by grant funds from the Uehara Memorial Foundation (to R. Tatsumi). Dr. A. Elgaabari was supported by a JSPS Postdoctoral Fellowship for Research in Japan (Standard FY 2022; P22389) during the course of this research. The funders had no role in study design, data collection and analysis, decision to publish, or preparation of the manuscript, and did not provide support in the form of salaries for any authors.

## CONFLICT OF INTEREST STATEMENT

The authors declare that the research was conducted in the absence of any commercial or financial relationships that could be construed as a potential conflict of interest.

## Supporting information


Data S1:

Figure S1.

Figure S2.

Figure S3.

Figure S4.

Figure S5.

Table S1.

## Data Availability

Data generated and analyzed in this study are included in the paper and the Supporting Information (Figures [Link], [Link], [Link], [Link], [Link] and legends), and are also available to interested person from the corresponding author upon reasonable request.
