# Supplementary material for: In vitro immuno‐prevention of nitration/dysfunction of myogenic stem cell activator HGF, towards developing a strategy for age‐related muscle atrophy
Source: Aging Cell. 2024 Sep 19;23(10):e14337. doi: 10.1111/acel.14337 (PMC11464115; doi:10.1111/acel.14337)
Supplement: Supplementary file 1 — Data S1: [file ACEL-23-e14337-s003.docx]

**SUPPORTING INFORMATION**

*Aging Cell*

Short Communication

**In vitro immuno-prevention of nitration/dysfunction of myogenic stem cell activator HGF, towards developing a strategy for age-related muscle atrophy**

Sakiho Tanaka^1^, Alaa Elgaabari^1,2^, Miyumi Seki^1^, So Kuwakado^3^, Kahona Zushi^1^,

Junri Miyamoto^1^, Shoko Sawano^4^, Wataru Mizunoya^5^, Kenshiro Ehara^1^,

Naruha Watanabe^1^, Yohei Ogawa^1^, Hikaru Imakyure^1^, Reina Fujimaru^1^, Rika Osaki^1^,

Kazuki Shitamitsu^1^, Kaoru Mizoguchi^1^, Tomoki Ushijima^1^, Takahiro Maeno^1^,

Takashi Nakashima^6^, Takahiro Suzuki^1^, Mako Nakamura^1^,

Judy E. Anderson^7^, Ryuichi Tatsumi^1,^*

^1^Department of Animal and Marine Bioresource Sciences, Graduate School of Agriculture, Kyushu University, West Zone 5, Motooka 744, Nishi-ku, Fukuoka 819-0395, Japan

^2^Department of Physiology, Faculty of Veterinary Medicine, Kafrelsheikh University, El-Geish street, Kafrelsheikh 33516, Egypt

^3^Department of Orthopaedic Surgery, Faculty of Medical Sciences, Kyushu University, Maidashi 3-1-1, Higashi-ku, Fukuoka 812-8582, Japan

^4^Department of Food and Life Science, School of Life and Environmental Science, Azabu University, Sagamihara 252-5201, Japan

^5^Department of Animal Science and Biotechnology, School of Veterinary Medicine, Azabu University, Sagamihara 252-5201, Japan

^6^Department of Bioscience and Biotechnology, Graduate School of Agriculture, Kyushu University, West Zone 5, Motooka 744, Nishi-ku, Fukuoka 819-0395, Japan

^7^Department of Biological Sciences, Faculty of Science, University of Manitoba, Winnipeg, MB R3T 2N2, Canada

*Correspondence: Ryuichi Tatsumi (Email: [rtatsumi@agr.kyushu-u.ac.jp](mailto:rtatsumi@agr.kyushu-u.ac.jp))

**Contents:**

1. Methodology (the entire Materials and Methods section)

2. Supplementary Figures 1-5 (Figs. S1-S5) and Legends, and Table S1 attached separately

3. References cited in Supporting Information

**METHODOLOGY (the entire Materials and Methods section)**

**1. Materials**

Recombinant mouse HGF (2207-HG/CF; carrier-free; disulfide-linked heterodimer of α and β chains as the major form in the product) and human interleukin 6 (IL-6; 206-IL) were purchased from R&D Systems (Minneapolis, USA). Peroxynitrite (P332) was purchased from Dojindo Laboratories (Kumamoto, Japan). Horseradish peroxidase (HRP)-labeled monoclonal anti-nitrotyrosine (clone 39B6; sc-32757 HRP), and HRP-labeled monoclonal anti-human HGF α-chain Abs (clone H-10, sc-374422 HRP) were from Santa Cruz Biotechnology (Dallas, TX, USA).

Dulbecco's modiﬁed Eagle's medium (DMEM; 31600-034), normal horse serum (HS; 16050-122), antibiotic-antimycotic (15240-062), gentamicin (15710-064), and hybridoma SFM medium (12300067) were purchased from Invitrogen (Grand Island, NY, USA). Poly-L-lysine (P9155), bovine plasma ﬁbronectin (F1141), protease type XIV (P5147), 5-bromo-2'-deoxyuridine (BrdU; B5002), HRP-labeled goat anti-mouse IgG Ab (A4416), 3,3'-diaminobenzidine (DAB; D5637), bovine serum albumin (BSA; A4503), hypoxanthine (H9377), and aminopterin (A3411) were all from Sigma-Aldrich (St. Louis, MO, USA).

Recombinant mouse HGFR/c-met Fc chimera (7065-ME; carrier-free), biotinylated polyclonal anti-human HGF Ab (BAF294; antigen affinity purified), streptavidin-HRP conjugate (4800-30-06), and tetramethyl benzidine (TMB) substrate solution (DY999) were purchased from R&D systems. Recombinant human NK1 (22.5 kDa; 32Q-210E with an additional sequence CHHHHHH-PRAAAVKSP at *N*-terminal) was a kind gift from TORAY (Tokyo, Japan). HRP-labeled rabbit anti-rat IgG (ab6734) and goat anti-rat IgG Fc (ab97090; immunogen-affinity purified) were purchased from Abcam (Cambridge, MA, USA). Amersham enhanced chemiluminescence (ECL) detection kit (PRN2106) and nitrocellulose membranes (10600048) were purchased from GE Healthcare (Little Chalfont, UK). Purified normal rat IgG (147-09521), keyhole limpet hemocyanin (KLH; 151233), and thymidine (205-08091) were from FUJIFILM Wako Pure Chemical (Osaka, Japan). Monoclonal anti-BrdU (clone G3G4) and anti-desmin Abs (clone D3) were purchased from Developmental Studies Hybridoma Bank (Iowa City, IA, USA).

**2. Animal care and use**

All experiments involving animals were conducted in strict accordance with the recommendations in the Guidelines for Proper Conduct of Animal Experiments published by the Science Council of Japan and ethics approvals from the Kyushu University Institutional Review Board (approval No. A20-014 and A22-082).

Sex as a biological variable: we utilized male rats just to isolate a larger number of cells, satellite cells from muscle tissue and B cells from the lymph nodes, per individual animal, respectively (Figures 2A,B and S1); sex was not considered as a biological variable.

**3. Production of anti-Y198-HGF monoclonal antibodies**

Monoclonal anti-Y198-HGF antibodies were raised against synthetic peptide containing nitrated tyrosine residue 198 (FTSNPEVR _nitro_Y_198_ EV) in a 2018-2019 period by a rat lymph node method established by Sado et al. (1995), as described previously (Elgaabari et al., 2024). Briefly, a cysteine residue was added to the *N*-terminal of peptide to enable conjugation with a carrier protein of BSA or KLH. Hind footpads of male WKY/Izm rats received an injection of KLH-conjugated nitrated Y198 peptide. B cells from the lymph nodes were fused with myeloma SP2/0-Ag14 cells; the obtained hybridoma cells were seeded onto 96-well plates and cultured in HAT selection medium, hybridoma SFM medium additionally containing 10% fetal bovine serum (FBS), 1 ng/ml recombinant human IL-6, 100 μM hypoxanthine, 0.4 μM aminopterin, and 16 μM thymidine.

Conditioned media from hybridoma cultures were screened by ELISA against BSA-conjugated nitrated/non-titrated Y198 and Y250 peptides and against nitrated/non-nitrated whole HGF protein immobilized to 96-well microplates (Figure S1, panels A and B, respectively). Immunoreactivity was visualized by HRP-conjugated anti-rat IgG Ab and TMB substrate, and one hybridoma positive for both nitrated and non-nitrated Y198 peptides/HGF and negative for nitrated Y250 peptide was selected (1H4). Hybridoma clones were established for clonality and unique immuno-specificity by cloning steps consisting of limiting dilution culture, ELISA of nitrated/non-nitrated peptides and HGF protein, and Western blotting of SDS-treated nitrated/non-nitrated HGF (Figure 1 and Figure S1 panel C), and were designated IH41C10 and 1H42F4 (abbreviated here as 1C10 and 2F4, respectively).

Immunoglobulin isotypes of clones 1C10 and 2F4 were both determined to be rat IgG2a(κ), subclass G2a for heavy chains and kappa for light chains by Rat Monoclonal Antibody Isotyping Test Kit (RMT1; Bio-Rad Lab., Berkeley, CA, USA).

Fab and Fc segments of rat 1C10 IgG were prepared by immobilized-papain digestion (Pierce Fab Preparation Kit; Thermo Fisher Scientific, Waltham, MA, USA) and protein L affinity spin-column chromatography (Rapid SPiN L; ProteNova, Higashikagawa, Japan); flow-through (Fc fragment) from protein L column was collected and Fab fragment was eluated with 0.1 M glycine-HCl, pH 2.7 followed by neutralization with 1 M Tris-HCl, pH 8.5. Protein purity was monitored by SDS-PAGE using 4-20% polyacrylamide gradient gels under non-reducing conditions.

Concentrations of the monoclonal antibodies produced (whole IgG, and Fab and Fc fragments) were determined by microdose-spectrophotometry (NanoDrop Lite; Thermo Fisher Scientific) at 280 nm of wavelength and 13.7 (L/gm-cm) of absorbance mass absorption coefficient for IgG.

**4. Immuno-pretreatment of HGF and nitration**

Recombinant mouse HGF (carrier protein-free) was pre-treated with anti-nitroY198-HGF mAbs (1C10 and 2F4 in PBS) at mole ratios (HGF : mAb) of 1:0, 1:0.025, 1:0.05, 1:0.1, 1:0.5, 1:1, and 1:5 (or with rat control IgG at 1:0.4) at pH 7.2, 25˚C for 30 min. Normal rat IgG was used as the control. HGF was evaluated for susceptibility to tyrosine nitration by incubating with peroxynitrite for 30 min under physiological conditions (pH 7.4, 25˚C) and by subsequent Western blotting to detect nitrotyrosines (see Figure 1c for the experimental design) (Elgaabari et al., 2024); peroxynitrite (0.1 M stock solution in 0.3 M NaOH-NaCl) was diluted to less than 1/100 with ice-cold water, just prior to quick exposure to HGF in PBS by vortexing at mole ratio (HGF : peroxynitrite) of 1:500. HGF with solvent alone (without adding peroxynitrite) was assigned as the control (1:0; see Figure 1d).

**5. ECL-Western blotting**

Tyrosine nitration was visualized by Western blotting of peroxynitrite-treated HGF according to Elgaabari et al. (2024). Briefly, proteins were subjected to sodium dodecyl sulfate-10% polyacrylamide gel electrophoresis (SDS-10% PAGE) under reducing conditions in Laemmli’s buffer system followed by transfer to nitrocellulose membranes (Tatsumi et al., 1998). Blots were incubated overnight at 4°C with HRP-conjugated anti-nitrotyrosine mouse mAb (1:2500 dilution in CanGetSignal solution 1; NKB-101, Toyobo, Osaka, Japan), followed by ECL-detection on a FUSION SOLO.7S.EDGE imaging system (Vilber Lourmat, Marne-la-Vallée, France). Subsequently, the blots proceeded through a second round of immunodetection by agitating for 45 min at 50˚C in stripping buffer (2% SDS, 0.8% β-mercaptoethanol (βME), and 62.5 mM Tris-HCl, pH 6.8) (Elgaabari et al., 2022, 2024) followed by re-probing with HRP-labeled anti-HGF *N*-terminal domain mAb (1:2500 dilution in CanGetSignal solution 1) to monitor HGF α-chain amount on the blots as loading controls. Further immunoblotting analysis of the nitration level of HGF was performed in the same manner using anti-nitroY198/Y250-HGF mAbs (1:2500 dilution; clones 1C6 and 2C3 raised in-house to visualize specifically nitroY198-HGF and nitroY250-HGF, respectively; see Elgaabari et al., 2024) and HRP-labeled secondary antibody (immunogen-affinity purified; 1:5000 dilution in CanGetSignal solution 2). In experiments to examine the effect of 1C10-Fab and -Fc segments, HRP-conjugated anti-rat IgG Fc specific secondary antibody was used.

Densitograms of nitrated HGF α-chain bands were measured by ImageJ 1.34 s (originally developed by Dr. Wayne Rasband, National Institutes of Health, Bethesda, MD, USA) and normalized with total HGF α-chain (nitrated and non-nitrated HGF α-chain; see Figure S2).

**6. Satellite cell preparation and activation assay**

Satellite cells were isolated from the upper hindlimb and back muscles of adult male Sprague-Dawley rats according to Allen et al. (1997) with a slight modification (Tatsumi et al., 2006a). Briefly, muscles were collected, trimmed of connective tissue and fat, minced with scissors, then digested with 1.25 mg/ml of protease type XIV for 1 h at 37˚C. Cells were separated from muscle tissue debris by differential centrifugation and filtration (nylon cell strainers) prior to a final centrifugation step at 1500 x g for 3 min, then suspended in DMEM containing 10% normal horse serum (HS), 1% antibiotic-antimycotic mixture, and 0.5% gentamicin (DMEM-10% HS), and plated on poly-L-lysine and fibronectin-coated plates. Cultures were maintained in a humidified atmosphere of 5% CO_2_ at 37˚C for 24 h and then incubated for the next 24-h period in DMEM-10% HS additionally containing recombinant HGF that was treated with peroxynitrite with/without 1C10/2F4 mAb pre-treatment (see Figure 2a).

Cultures were pulse-labeled with 10 µM BrdU in DMEM-10% HS for the final 2 h period of 48-h in culture, followed by immunocytochemistry for detection of BrdU using G3G4 anti-BrdU mAb (1:100 dilution) and HRP-conjugated anti-mouse IgG Ab (1:500 dilution) according to Tatsumi et al. (1998). The mean percentage of BrdU-labeled cells for three cultures per treatment was used as an indicator of activation (entry into the cell cycle) and the subsequent proliferation activity of plated satellite cells. Companion satellite-cell cultures, prepared at the same time, were immunostained at 30-h post-plating with D3 anti-desmin mAb to determine the percentage of myogenic cells present (Allen et al., 1995; Tatsumi et al., 1998); cultures with less than 95% DAB-positive cells were not used for experiments.

**7. c-Met binding assay**

According to Tatsumi et al. (2002) with some minor modifications (Elgaabari et al., 2024), 1C10/peroxynitrite-treated HGF was evaluated for *in vitro* c-met binding affinity. Briefly, 96-well ELISA microplates were coated with HGFR/c-MET Fc chimera (50 ng/well), blocked with 1% BSA, 5% sucrose, and 0.05% sodium azide in PBS, and then incubated for 2 h at 37˚C with HGF samples. Plates were subsequently washed with 0.1% polyethylene sorbitan monolaurate (Tween20)-Tris-buffered saline (TTBS), immediately fixed with cold 3.7% paraformaldehyde in PBS, and re‑treated with the blocking solution overnight at 4˚C. The binding of HGF to c-met was detected with biotinylated anti-HGF polyclonal Ab (1:500 dilution in the 0.1% BSA-TTBS for 3 h at 25˚C), TACS streptavidin-HRP conjugate (1:1600 dilution for 20 min), and TMB substrate solution, followed by coloration stopping and optical density measurements at wavelengths of 450 and 545 nm.

**8. RT-qPCR**

Total RNA was isolated from rat satellite cell cultures by a regular ISOGEN II protocol (Nippon Gene, Tokyo, Japan). cDNA was synthesized from total RNA by a ReverTra Ace qPCR RT Kit (TOYOBO, Osaka, Japan). mRNA expression of MyoD (NCBI RefSeq accession no. NM_176079.1) and myogenin (NM_017115.2) was monitored by real-time quantitative PCR using Roche LightCycler 96 (Mannheim, Germany) run under the SYBER GREEN detection format standardized with hypoxanthine guanine phosphoribosyl transferase (HPRT, NM_012583.2). The primer sets were designed by the Roche ProbeFinder (version 2.35 for rat) with an intron-spanning assay for rat MyoD (amplicon 69 nt), myogenin (101 nt), and HPRT (61 nt) as shown in Table S1 (Suzuki et al., 2013; Suzuki et al., 2021; Maeno et al., 2023). Annealing temperature was set to 60°C in all cases (Figure S4 panel B).

**9. Statistical analyses**

Student's t-tests were employed for statistical analysis of experimental results using Microsoft Excel X for Windows. Data are represented as mean ± standard error of the mean (SEM). The level of significance was set to *p* < 0.05 throughout this study and statistically significant differences between two groups at *p* < 0.05 and *p* < 0.01 are indicated on graphs by (*) and (**), respectively. The results are representative examples of more than two or three independent experiments.

**LEGENDS TO SUPPLEMENTARY FIGURES**

**FIGURE S1. Generation of anti-Y198-HGF monoclonal antibodies (screening and cloning).**

**(A)** Monoclonal antibodies were raised against a sequence that includes nitrated Y198, FTSNPEVR _nitro_Y_198_ EV, as described before (Elgaabari et al., 2024). B cells from the lymph nodes were fused with myeloma SP2/0-Ag14 cells; conditioned media from hybridoma cultures were first screened by ELISA against BSA-conjugated nitrated/non-titrated Y198 peptides and nitrated Y250 peptide immobilized to 96-well microplates. Immunoreactivity was visualized by HRP-conjugated anti-rat IgG antibody (Ab) and TMB substrate.

**(B)** The second ELISA-screening was performed by assessing the positive-reactivity to both nitrated HGF (recombinant full-length HGF treated with peroxynitrite) and non-nitrated HGF; hybridoma 1H4 was obtained (black-circled; *upper-half a*). Hybridomas 3A1 and 8B7 for the nitrated Y198 peptide (red-circled; *upper-half a*) and 3C3 and 6B8 for the nitrated Y250 peptide (blue-circled; *lower-half b*) were served as controls that show the immuno-specificity for nitrated HGF. Reproduced from Figure S4, panels A and C (Elgaabari et al*.*, 2024) with some modifications under the permission from John Wiley & Sons Ltd. and The Anatomical Society.

**(C)** Single-cloning of hybridomas by limiting dilution cultures. Selected clones from 1H4 were examined for the immuno-reactivity to establish unique hybridoma clones IH41C10 and IH42F4 (abbreviated as 1C10 and 2F4, respectively, in this study) that recognize both nitrated and non-nitrated Y198 peptides.

**FIGURE S2. Densitometric analysis for Figure 1D.**

Densitograms of nitrated HGF bands shown in Figure 1d (Western blotting) were determined by ImageJ densitometry normalized with total HGF α-chain (nitrated and non-nitrated HGF that were visualized by anti-HGF α-chain mAb) and presented as relative to the control, non-nitrated regular HGF (far-left bars, 1:0 1C10/2F4-treatment groups).

**FIGURE S3. c-Met binding activity of HGF-NK1 segment.**

Evaluated by sandwich ELISA-like assay on c-met-Fc chimera as in Figure 2, panels c and d (see photos of the quadruple assay shown in upper column). Optical absorbance_450-535 nm_ was presented by subtracting the value of the negative control without NK1 (*far-left bar*). Left *black-bar*, positive control (non-nitrated HGF); right *black-bar*, control non-nitrated NK1. *Gray bar*, nitrated NK1 (1:500 peroxynitrite treatment) served as a control showing that the c-met binding activity was decreased down to a level comparative to the negative control upon Y198 nitration. Bars represent mean ± SEM with different lower-case alphabets showing significant differences at *p <* 0.01. Note that there is a significant difference in binding affinity for the receptor c-met between HGF and NK1 that encompasses Y198 in K1 domain (see *black bars*).

**FIGURE S4. Cell proliferation activity of HGF that was pre-treated with 1C10.**

**(A)** The experimental scheme for assays in culture. Primary cultures of rat satellite cells received 5 ng/ml HGF (control and 1C10-pretreated at 1:5 mole ratio relative to HGF for 30 min at 25℃, as shown in Figure 2b, *bars b* and *c*, respectively) in DMEM-10% HS (pH 7.2) for 24 h beginning at 24-h post-plating and maintained for the next 24-h period in media without HGF.

**(B)** Cell proliferation response assayed by RT-qPCR for mRNA expression levels of MyoD and myogenin at 24-72 h post-plating. Rat satellite cells were further separated from muscle tissue debris by Percoll gradient centrifugation prior to a plating step on 24-well cluster dishes at 3.5 x 10^5^ cells/well in DMEM-10% HS. *Open bars a*, negative control untreated-cultures in DMEM-10% HS; *black-solid bars b*, positive control cultures with 5 ng/ml recombinant HGF for 24 h beginning 24-h post-plating; *red bars c*, cultures with 5 ng/ml HGF that received pre-treatment with 1C10 (at 1:5 mole ratio relative to HGF). Bars depict the mean and SEM (3 wells per group) as arbitrary units relative to the mean of the 24-h negative controls. Significant differences from the 24-h negative controls at *p* < 0.05 and *p* < 0.01 are indicated by (*) and (**), respectively. *NS*, not significant at *p <* 0.05; note that there is no significant difference in the mRNA expression levels of MyoD and myogenin between control HGF and 1C10-pretreated HGF cultures at 48-h and 72-h time-points, indicating that mAb 1C10-bound HGF can normally stimulate cell activation and proliferation followed by the early differentiation. Insets, representative micrographs (phase contrast) of cultures at each time-point.

**(C)** Reactivity of 1C10 to ECM-bound HGF in rat satellite cell culture. At 24-h (*inset*) and 48-h post-plating, live cells were incubated with fluorescein-labeled 1C10 in culture medium (DMEM-10% HS) for 60 min at 37℃, and observed directly under a Leica DMI6000B-AFC fluorescence microscope without a coverslip (immuno-fluorescence merged with bright-field images). It is noteworthy that 1C10-negative cells appear to be satellite cells derived from types I and IIb myofibers, which have exhibited negative or faint localization of extracellular HGF as demonstrated previously by direct immunofluorescence of cryosections of rat lower hindlimb muscles (gastrocnemius, plantaris, soleus, extensor digitorum longus, and tibialis anterior muscles; Elgaabari et al., 2024).

**FIGURE S5. Effect of pre-treatment with 1C10 and 2F4 on HGF nitration (induced at an elevated peroxynitrite ratio 1:2000).**

Recombinant HGF was incubated with 1C10 and 2F4 in mole ratio ranges depicted for 30 min prior to a peroxynitrite-protocol (at 1:2000 mole ratio relative to HGF, four times higher than that in Figure 1d). Visualized by HRPO-labeled anti-nitrotyrosine (anti-nitroY) mAb in Western blotting format normalized by total HGF α-chain (detected with HRP-labeled anti-HGF α-chain mAb after the stripping step as described in Figure 1b). MW-STD, MagicMark molecular weight standards.

**REFERENCES**

1. Maeno, T., Arimatsu, R., Ojima, K., Yamaya, Y., Imakyure, H., Watanabe, N., Komiya, K., Kobayashi, K., Nakamura, M., Nishimura, T., Tatsumi, R., & Suzuki, T. (2023). Netrin-4 synthesized in satellite cell-derived myoblasts stimulates autonomous fusion. *Exp Cell Res*, 430(1), e113698.
2. https://doi.org/10.1016/j.yexcr.2023.113698
3. Sado, Y., Kagawa, M., Kishiro, Y., Sugihara, K., Naito, I., Seyer, J. M., Sugimoto, M., Oohashi, T., & Ninomiya, Y. (1995). Establishment by the rat lymph node method of epitope-defined monoclonal antibodies recognizing the six different α chains of human type IV collagen. *Histochem Cell Biol*, 104(4), 267-275.
4. https://doi.org/10.1007/BF01464322
5. Suzuki, T., Do, M.-K. Q., Sato, Y., Ojima, K., Hara, M., Mizunoya, W., Nakamura, M., Furuse, M., Ikeuchi, Y., Anderson, J. E., & Tatsumi, R. (2013). Comparative analysis of semaphorin 3A in soleus and EDL muscle satellite cells in vitro toward understanding its role in modulating myogenin expression. *Int J Biochem Cell Biol*, 45(2), 476-482.

https://doi.org/10.1016/j.biocel.2012.10.003

1. Suzuki, T., Mori, A.; Maeno, T., Arimatsu, R., Ichimura, E., Nishi, Y., Hisaeda, K., Yamaya, Y., Kobayashi, K., Nakamura, M., Tatsumi, R., Ojima, K., & Nishimura, T. (2021). Abundant synthesis of netrin-1 in satellite cell-derived myoblasts isolated from EDL rather than soleus muscle regulates fast-type myotube formation. *Int J Mol Sci*, 22(9), e4499.

https://doi.org/10.3390/ijms22094499
