# Supplementary figures and images for: In vitro immuno‐prevention of nitration/dysfunction of myogenic stem cell activator HGF, towards developing a strategy for age‐related muscle atrophy
Source: Aging Cell. 2024 Sep 19;23(10):e14337. doi: 10.1111/acel.14337 (PMC11464115; doi:10.1111/acel.14337)

Supplemental Materials

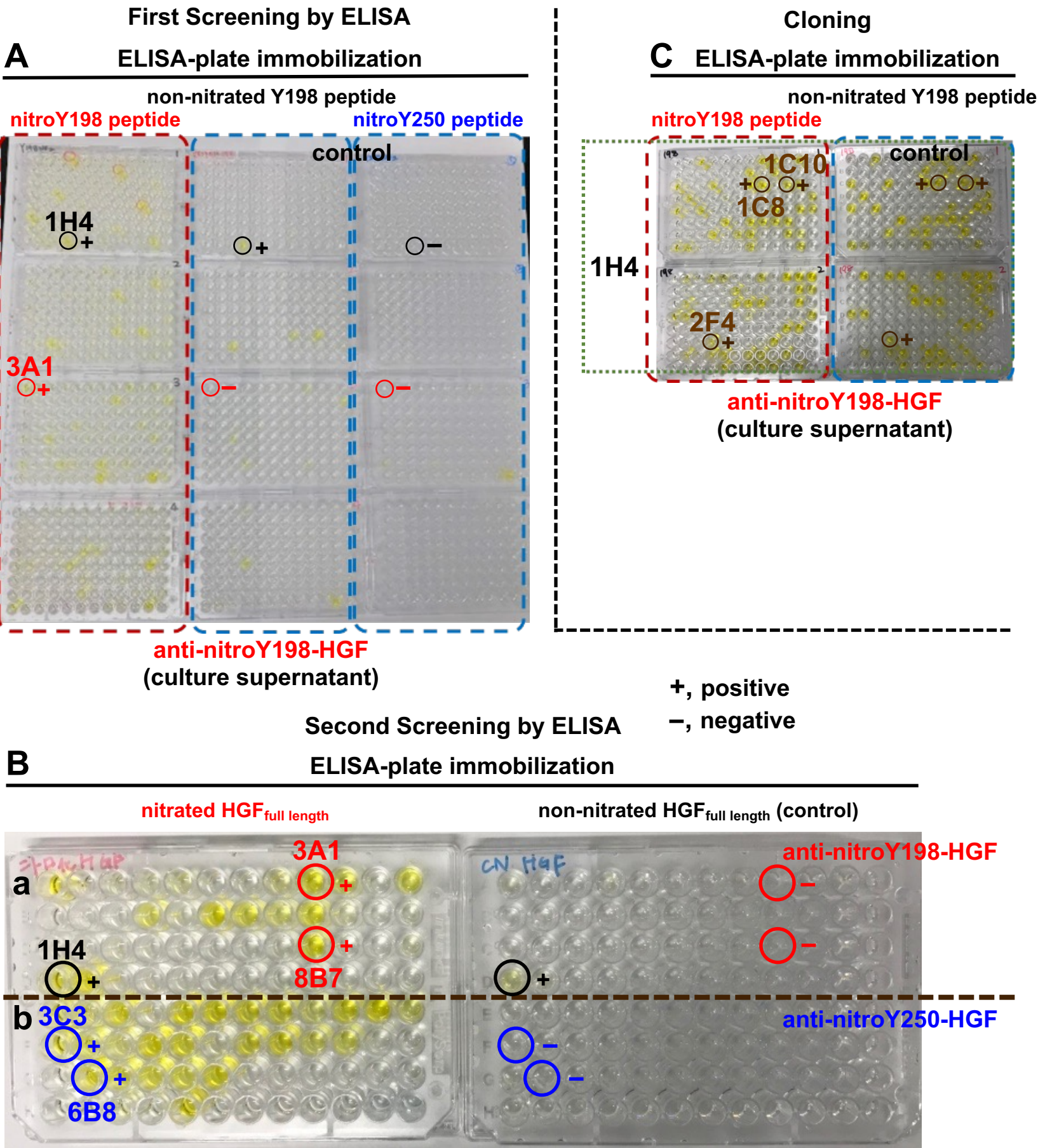

Supplementary Fig. 1 (Fig. S1), Tanaka *et al.*

Supplement: Supplementary file 2 — Figure S1. [file ACEL-23-e14337-s002.pdf]

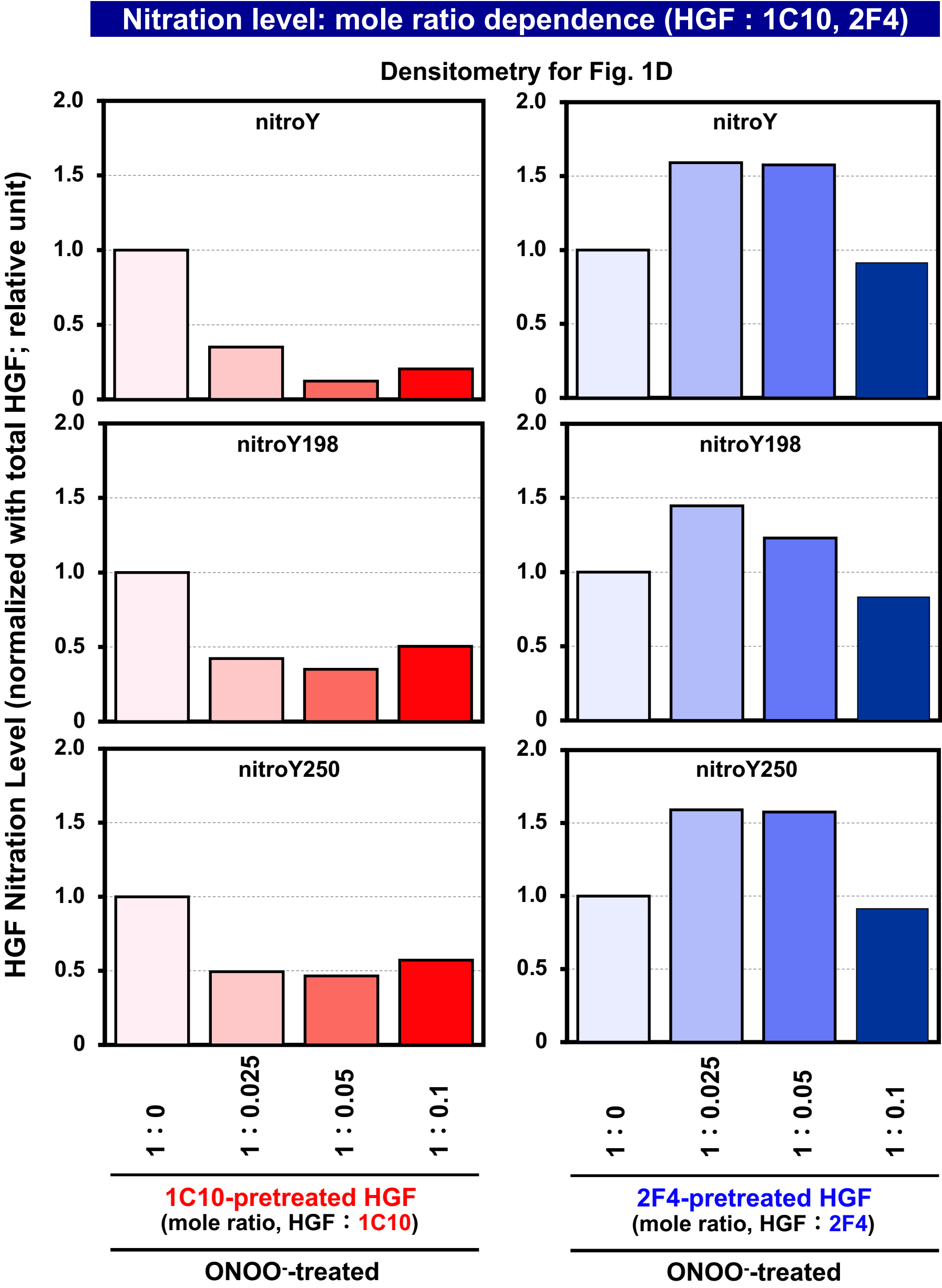

Supplementary Fig. 2 (Fig. S2), Tanaka *et al.*

Supplement: Supplementary file 3 — Figure S2. [file ACEL-23-e14337-s001.pdf]

## Supplemental Materials

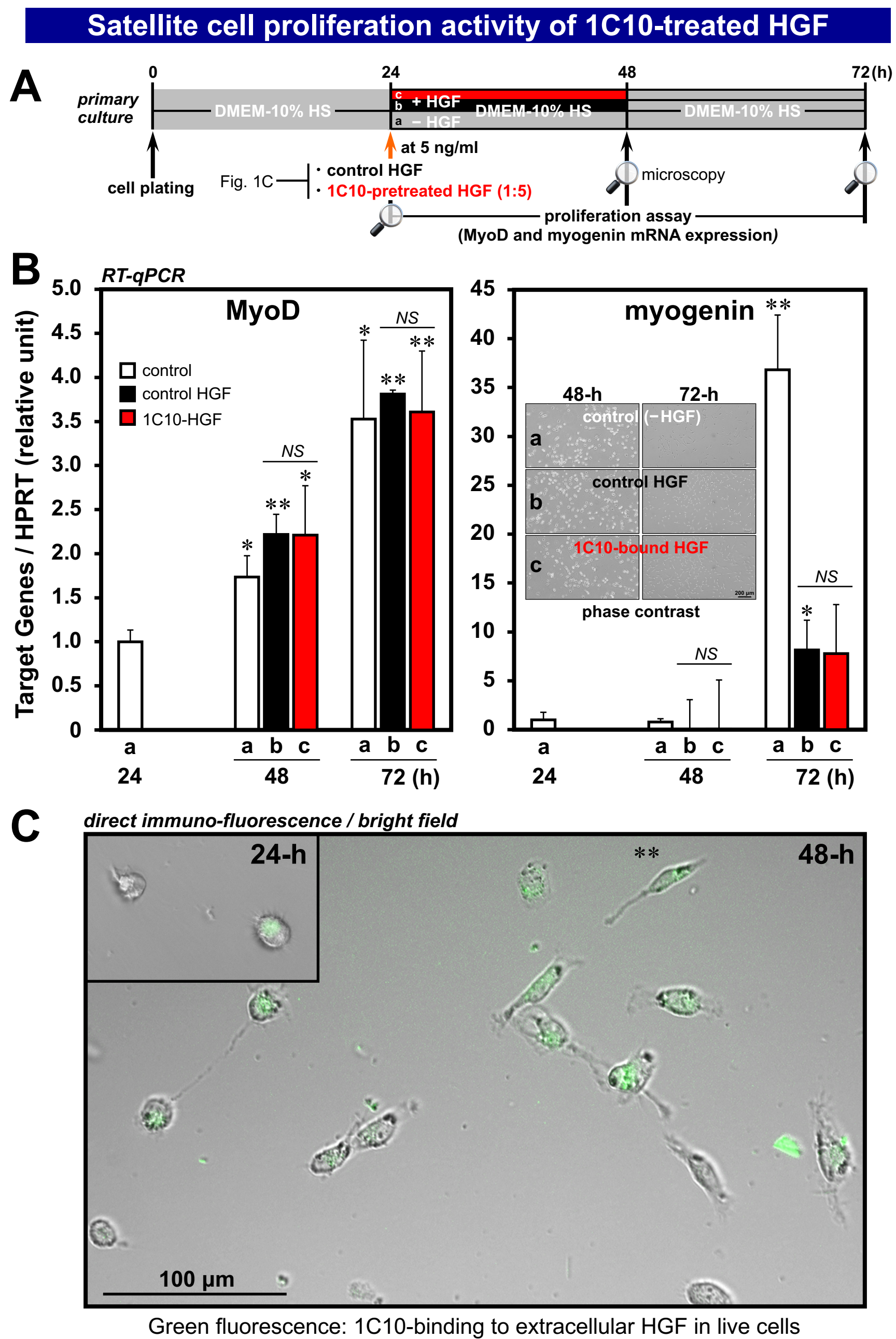

Supplementary Fig. 4 (Fig. S4), Tanaka *et al.*

Supplement: Supplementary file 5 — Figure S4. [file ACEL-23-e14337-s005.pdf]

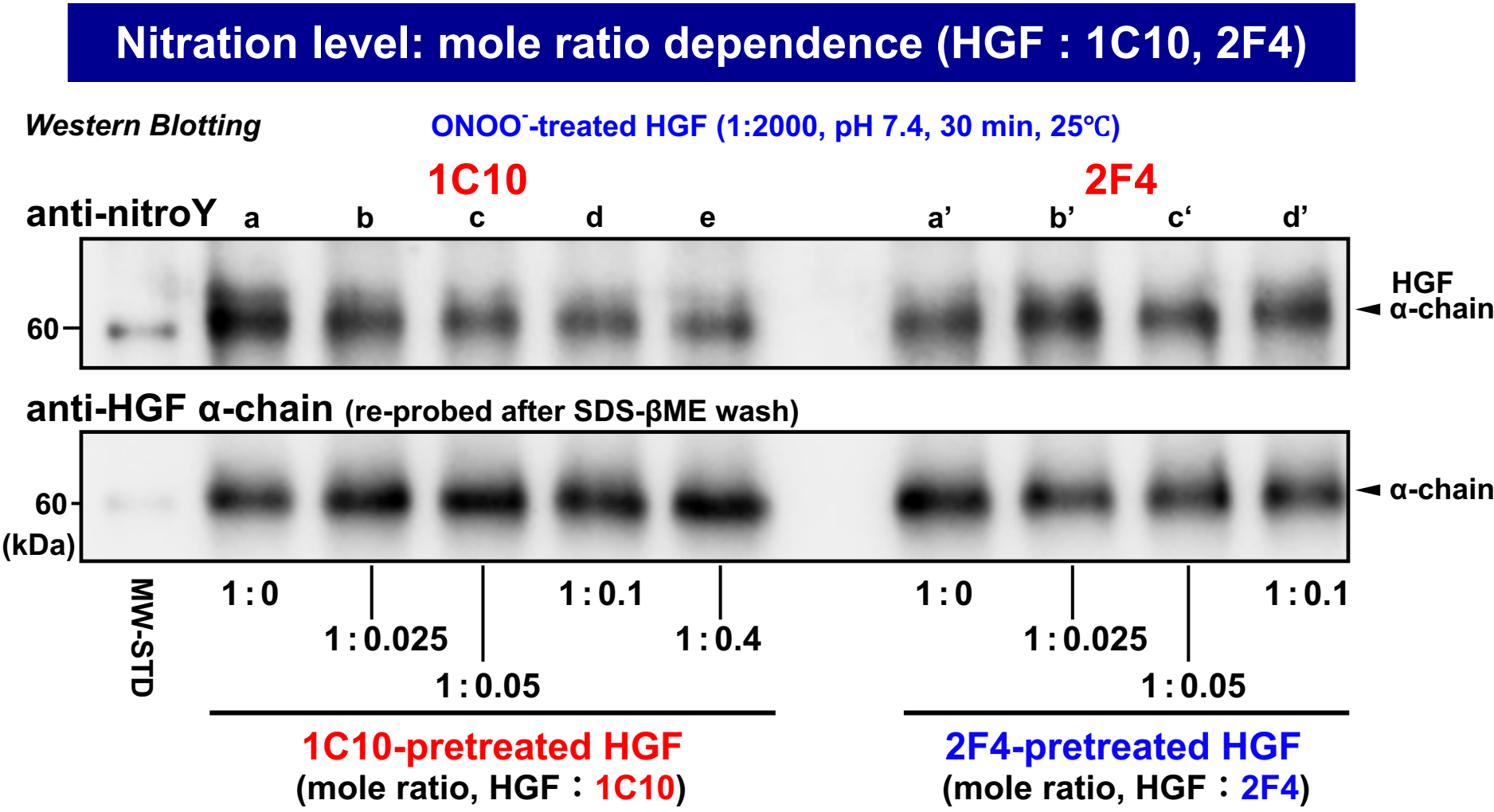

Supplementary Fig. 5 (Fig. S5), Tanaka *et al.*

Supplement: Supplementary file 6 — Figure S5. [file ACEL-23-e14337-s007.pdf]
