# Supplementary material for: In vitro immuno‐prevention of nitration/dysfunction of myogenic stem cell activator HGF, towards developing a strategy for age‐related muscle atrophy
Source: Aging Cell. 2024 Sep 19;23(10):e14337. doi: 10.1111/acel.14337 (PMC11464115; doi:10.1111/acel.14337)
Supplement: Supplementary file 4 — Figure S3. [file ACEL-23-e14337-s006.pdf]

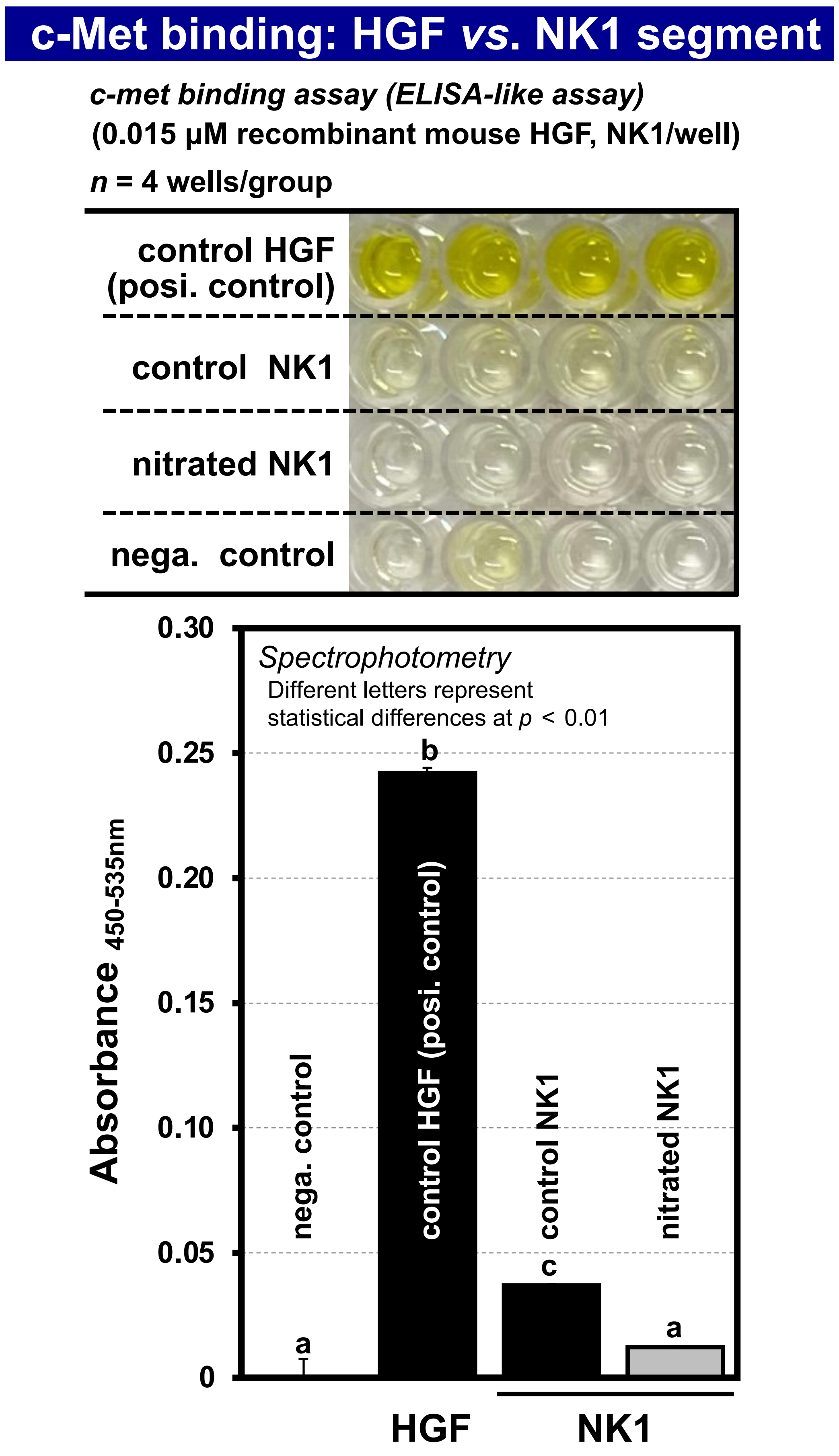

Supplementary Fig. 3 (Fig. S3), Tanaka *et al.*
