# Supplementary material for: In vitro immuno‐prevention of nitration/dysfunction of myogenic stem cell activator HGF, towards developing a strategy for age‐related muscle atrophy
Source: Aging Cell. 2024 Sep 19;23(10):e14337. doi: 10.1111/acel.14337 (PMC11464115; doi:10.1111/acel.14337)
Supplement: Supplementary file 7 — Table S1. [file ACEL-23-e14337-s004.pdf]

Table S1. PCR primer sets for rat MyoD, myogenin, and HPRT.

Primer name	Primer sequence (5' to 3') forward	Primer sequence (5' to 3') reverse
MyoD	CCCTGTTGTTTGTGGAGACA	CTGTGGGAAAGAGTGGGTGT
myogenin	CTACAGGCCTTGCTCAGCTC	TGGGAGTTGCATTCACTGG
HPRT	GACCGGTTCTGTCATGTCG	ACCTGGTTCATCATCACTAATCAC
